# CA19-9-related macrophage polarization drives poor prognosis in HCC after immune checkpoint inhibitor treatment

**DOI:** 10.3389/fonc.2024.1528138

**Published:** 2025-01-10

**Authors:** Tingting Du, Jialin Zou, Yunying Yang, Honghui Xie, Hui Pang, Wenquan Zhuang, Shutong Wang, Guangyan Wei

**Affiliations:** ^1^ Department of Radiation Oncology, The First Affiliated Hospital, Sun Yat-sen University, Guangzhou, China; ^2^ Department of Anorectal Surgery, Shenzhen Longgang Central Hospital, Shenzhen, China; ^3^ Department of Gynecology, Lingshan County People’s Hospital, Qinzhou, China; ^4^ Management Evaluation Section, The First Affiliated Hospital, Sun Yat-sen University, Guangzhou, China; ^5^ Department of Interventional Radiology, The First Affiliated Hospital, Sun Yat-sen University, Guangzhou, China; ^6^ Center of Hepato-Pancreato-Biliary Surgery, The First Affiliated Hospital, Sun Yat-sen University, Guangzhou, China

**Keywords:** HCC, CA19-9, immunotherapy, tumor microenvironment, macrophages

## Abstract

**Background:**

Elevated levels of carbohydrate antigen 19-9 (CA19-9) levels are known to worsen outcomes in various tumors by influencing immune responses. However, the role of CA19-9 in immunotherapy for hepatocellular carcinoma (HCC) remains poorly understood.

**Methods:**

This study included 621 patients treated with anti-PD-1/PD-L1 treatment at the First Affiliated Hospital of Sun Yat-sen University from January 2017 to March 2023. During immunotherapy, CA19-9 levels were measured and classified as either elevated (≥35 U/mL) or normal (<35 U/mL) for clinical analysis.

**Results:**

Patients with elevated CA19-9 levels had significantly worse progression-free survival (PFS) and overall survival (OS). The 1-year and 2-year PFS rates were 53.3% and 29.1% in the normal CA19-9 group compared to 16.9% and 11.3% in the elevated group (*p* < 0.001). Similarly, the 1-year and 2-year OS rates were 90.5% and 75.5% in the normal group versus 64.0% and 36.5% in the elevated group (*p* < 0.001). Multivariate analysis confirmed CA19-9 was an independent prognostic factor for both PFS and OS. Bioinformatic analysis indicated that FUT3, a key gene in CA19-9 synthesis, correlated with increased macrophage infiltration. And increased M2 macrophage levels and reduced M1 macrophage levels were noted in HCC samples with elevated CA19-9 levels. Further *in vivo* experiments indicated blocking CA19-9 improved the efficacy of PD-1 treatment through inducing the M1-like polarization of macrophages.

**Conclusions:**

Our findings demonstrate that elevated CA19-9 levels during immunotherapy are associated with poor survival outcomes in HCC patients. These findings highlight the crucial role of CA19-9 in shaping the tumor immune environment, particularly through its effect on macrophage polarization, and suggest that targeting CA19-9 may improve immunotherapy outcomes.

## 1 Introduction

Hepatocellular carcinoma (HCC) is among the most prevalent cancers globally and is the third leading cause of cancer-related deaths (1). Despite rapid advancements in the diagnosis and treatment, HCC in approximately 25%–70% of patients is diagnosed at an advanced stage due to its sudden onset and swift progression (2). Thus, treatment options for the condition are often limited, and the cure rate remains low. To address these challenges, systematic antitumor therapies have been employed for managing intermediate and advanced HCC. The combination of immunotherapy and targeted therapy, developed in the past decade, has become the first-line treatment for HCC, effectively prolonging patient survival (3). Previous studies have demonstrated the effectiveness of immune checkpoint inhibitors (ICIs), such as nivolumab and pembrolizumab, when used either alone or in combination, in improving survival of patients with advanced HCC (4, 5). However, the efficacy of programmed death-1 (PD-1)/programmed cell death ligand 1 (PD-L1) inhibitors varies widely among individuals and is predominantly affected by the heterogeneity of the tumor microenvironment (TME). This heterogeneity can weaken immune responses and lead to resistance against immunotherapy (6). Due to this complexity, exploring predictive factors that can help identify patients with HCC who would benefit from immunotherapy becomes essential.

Although alpha-fetoprotein (AFP) is the most commonly used biomarker for HCC, its role as a prognostic biomarker for immunotherapy remains controversial. Studies indicate that AFP levels <400 µg/L prior to PD-1 treatment are associated with increased PR or CR rates and reduced disease progression (PD). However, data from CheckMate 459 suggest that patients with baseline AFP levels >400 µg/L treated with Nivolumab exhibited longer overall survival (OS) (7, 8). Therefore, AFP is not an ideal predictive factor for immunotherapy outcomes in HCC.

Other markers, such as protein induced by carbohydrate antigen 19-9 (CA19-9), carcinoembryonic antigen (CEA), and carbohydrate antigen 125(CA125), have attracted attention for their diagnostic and prognostic relevance (9–11).

Among these, CA19-9, also known as Sialyl Lewis-a, is a sialylated glycoprotein synthesized on cell surfaces through enzymatic reactions (12). Sialylated glycans makes HCC cells have anti-adhesion characteristics and enhances the mobility and invasiveness of tumors (13). Studies have shown that desialylation of malignant tumors can enhance cellular immunotherapy by disrupting the interaction between sialoglycans and inhibitory receptors Siglec-5 and Siglec-10, thereby promoting infiltration and activation of induced pluripotent stem cell–derived chimeric antigen receptor-macrophages (CAR-iMac) (14). Additionally, targeted inhibition of CA19-9 reduces EGFR phosphorylation in pancreatic ductal cells, which enhances T cell infiltration and improves pancreatic cancer response to PD-1 and anti-CTLA4 therapies (15, 16). CA19-9, containing salivary acidification structure, may influence the prognosis of HCC immunotherapy by regulating immune cell infiltration within the tumor microenvironment (TME). Moreover, an elevated CA19-9 level is observed in approximately 30% of patients with HCC (17). Thus, CA19-9 may serve as a predictor of prognosis and response to immunotherapy. In particular, an elevated CA19-9 level before the initiation of immunotherapy was associated with a poor prognosis in patients with HCC (18). However, the implications of increased CA19-9 levels during immunotherapy in these patients remain unclear.

This study investigated the effect of immunotherapy on patients with HCC by analyzing their serum CA19-9 level and exploring its prognostic value. The findings of this study can shed light on how stratification by CA19-9 levels (serum CA19-9 ≥ 35 U/mL) can aid in identifying patients with HCC who might benefit from immunotherapy.

## 2 Materials and methods

### 2.1 Study population

From January 2017 to March 2023, a total of 611 HCC patients received immunotherapy (anti-PD-1 or anti-PD-L1) at the First Affiliated Hospital of Sun Yat-sen University.

**Figure 1:**
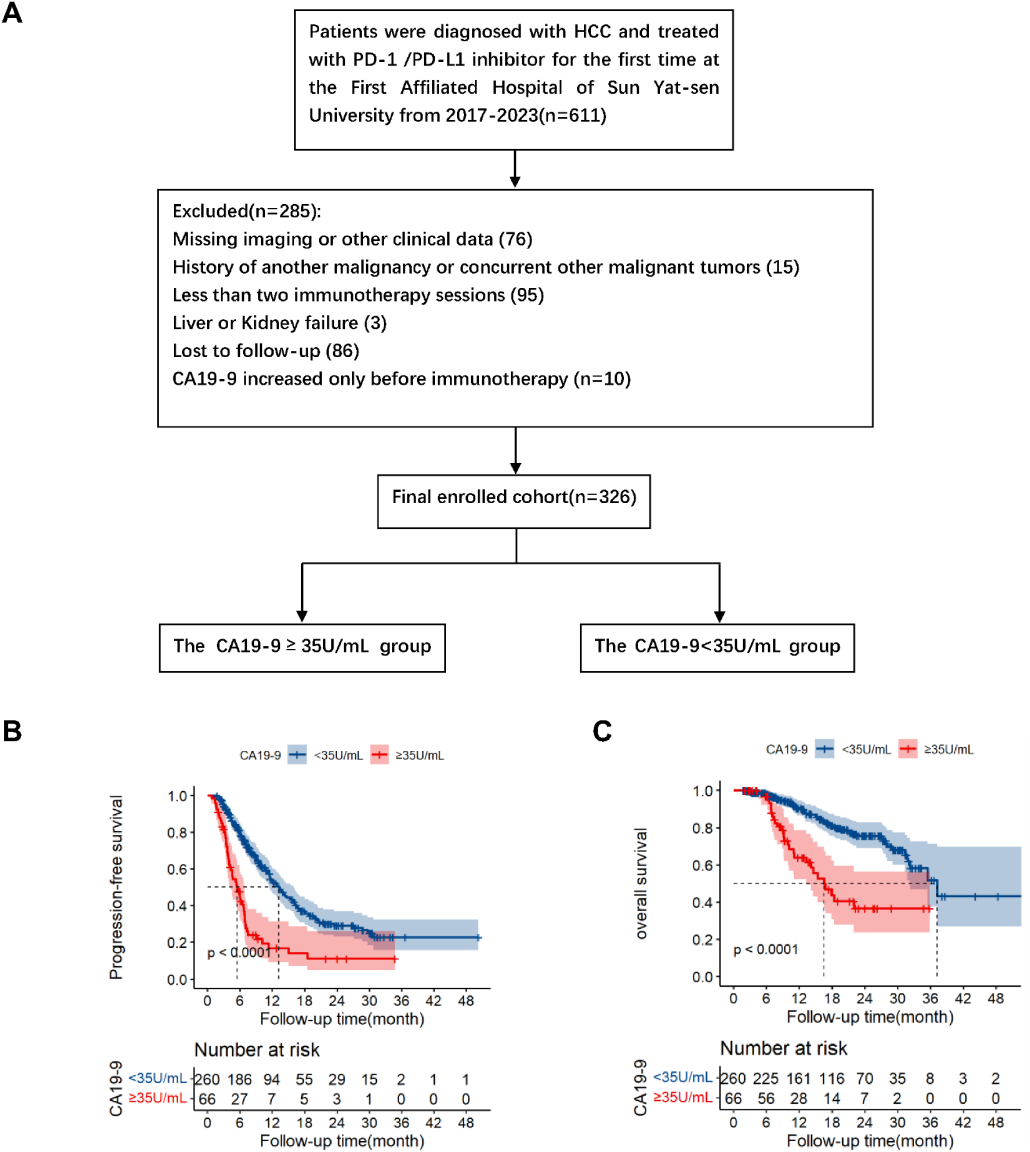
Flowchart of patient enrolment **(A)**. Kaplan–Meier survival analysis of progression-free survival **(B)** and overall survival **(C)** according to CA19-9 groups (log-rank tests, *p*<0.001).

The inclusion criteria were as follows: 1) diagnosed as hepatocellular carcinoma in BCLC C stage; 2) at least 2 cycles of immunotherapy and continuous immunotherapy according to the treatment plan; 3) Patients in the present study were categorized according to the post-immunotherapy change in CA19-9 levels, specifically, whether there was an increase or no increase (cut-off value: 35U/mL); 4) Complete clinical and follow-up data; 5) At least one measurable lesion as defined according to RECIST V1.1; 6) Child-Pugh class A, and Eastern Co-operative Oncology Group (ECOG) score ≤ 1. On the contrary, the exclusion criteria were as follows: 1) Combined with other malignant tumors; 2) Less than 2 course immunotherapy; 3) Lack of imaging, CA19-9, AST, ALT or other clinical data; 4) End-stage liver and kidney function; 5) Patients with CA19-9 increased before immunotherapy; 6) Lost to follow-up. The flow chart of the patient’s selection is shown in **Figure 1A**. According to the above criteria, a total of 326 patients were included in this study.

All patients signed an informed consent before immunotherapy. The Ethics Committee of the First Affiliated Hospital of Sun Yat-sen University approved the study, which was in line with the 2008 Helsinki Declaration of the World Medical Association.

### 2.2 Immune infiltration analysis

The TISIDB database was used to explored the relationship between FUT3 expression and immune subtypes or immunomodulators in HCC (19). The association between FUT3 and the relative enrichment score in 24 types of immune cells was carried out by the R package GSVA (20). The relationship in the infiltration of immunocytes between the high and low expression FUT3 groups was performed in Spearman’s correlation analysis.

### 2.3 Immunohistochemistry

IHC staining were performed in formalin-fixed, paraffin-embedded liver sections, as previously described (21). The primary antibodies (CD80 (1:100, Proteintech #66406) and CD206 (1:200, CST #24595)) were used in this study. Horse-radish peroxidase-conjugated anti-rat or anti-rabbit antibodies were used for detection, and images were captured using ZEISS Axio microscopy.

### 2.4 Cell lines

Mouse HCC cell line Hepa1-6 were obtained from the Cell Bank of the Type Culture Collection of the Chinese Academy of Sciences in January 2018. All cells were tested for Mycoplasma contamination using the single-step PCR method. All cells were cultured at 37°C and 5% CO2 in DMEM supplemented with 10% FBS (Gibco) and 1% penicillin–streptomycin.

### 2.5 Animal experiments

All wild-type C57BL/6 mice (5-6 weeks, male) were purchased from GemPharmatech (Nanjing, China). All animals were acclimatized for 1 week before experiments and housed in a specific pathogen-free environment with a 12-hour light-dark cycle and permitted ad libitum consumption of water and a standard chow diet unless otherwise stated. All mouse experiments were approved by the Institutional Care and Animal Use Committee of Sun Yat-sen University Cancer Center (20110M).

For subcutaneous tumor xenograft models, mice were randomly divided into different groups (n=3-5/each group) until the tumor volume reached approximately 80–130 mm^3^. Hepa1-6 cells (5 × 10^6^ cells resuspended in 150 μL PBS) were injected into the right flanks of the mice. Tumor growth was monitored every three days, and the mice were euthanized at the end of study using pentobarbital sodium (40 mg/kg). The tumor volume was measured using the following formula: 0.5 × (larger diameter) × (smaller diameter)^2^.

For drug treatment, 200 μg anti-PD1 antibody (Bio X Cell, West Lebanon, NH, USA) or anti-IgG (Bio X Cell) was injected intraperitoneally every three days. And 200 μg anti-CA19-9 (NS19-9) was daily intraperitoneal injected.

### 2.6 Flow cytometry

Tumor specimens were retrieved from euthanized murine subjects and processed into single-cell suspensions. Prior to antibody staining, single-cell suspensions were incubated with Fc receptor-blocking solutions to minimize non-specific binding. Specific antibodies were used to label the cells while maintained on ice.

For intracellular analysis, the Biolegend True-Nuclear™ Transcription Buffer Set (Catalog No. 424401) was employed. Following careful washing steps, both extracellular and intracellular antibodies were applied simultaneously to the cells. Fluorochrome-conjugated antibodies were sourced from reputable vendors including Biolegend, eBioscience, and BD Biosciences (CD45-BV421, CD45-APC-Cy7, FVD-ef506, CD3-APC, CD4-PE-Cy7, CD8-APC-Cy7, CD11b-APC, F4/80-FITC, MHC-II-PE, CD206-PE-Cy7, Ly6G-BV421, Ly6C-BV605). Data acquisition was performed using a BD flow cytometer, and subsequent analysis was conducted utilizing FlowJo software.

### 2.7 Data collection

All patients were collected blood samples before the first immunotherapy. All data included age, gender, maximal tumor diameter(MTD), number of tumors, portal vein tumor thrombus(PVTT), extrahepatic metastasis, hepatitis status, liver cirrhosis, CA19-9 levels, albumin (ALB), total bilirubin (TBIL), prothrombin time (PT). After that, CA19-9 levels should be collected as far as possible for each immunotherapy. Using the normal reference value established by our hospital as a benchmark, CA19-9 levels ≥35 U/mL are classified as the elevated group, whereas CA19-9 levels <35 U/mL are categorized as the normal group.

### 2.8 Follow-up

Usually, patients received anti-PD-1/PD-L1 treatment every 3 weeks, and the immunotherapy plan could be formulated and adjusted according to the tumor situation. Using computed tomography or magnetic resonance imaging, according to the response evaluation criteria in solid tumors (RECIST1.1), the tumor was evaluated every 2 treatment cycles after the first treatment and then it was evaluated every 3-6 months after half a year of the first anti-PD-1/PD-L1 treatment. The serum CA19-9 level was collected and updated synchronously. The follow-up data was based on inpatient or outpatient visits or telephone records, and the follow-up data collection was terminated on June 30, 2023.

### 2.9 Statistical analysis

The main research endpoint was overall survival (OS), which was defined as the time interval from the beginning of the first immunotherapy to the death of any cause or the survival of the last follow-up (deletion). The secondary research endpoint was progress-free survival (PFS), which was defined as the time from the beginning of the first treatment to disease progression (PD) or death or last follow-up (deletion).

Continuous variables used Wilcoxon rank sum test or student’s t-test, while classification variables used chi-square test or Fisher precision test to compare the correlation between CA19-9 level and clinical pathological characteristics. The Kaplan-Meier method was used to generate the OS and PFS curves, and the Log-rank test was used to analyze the differences between the two groups of different CA19-9 levels. Based on the COX proportional hazards model, the univariate and multivariate analysis of survival were carried out to confirm independent predictors for PFS and OS. Clinical pathological factors with *P*<0.05 in univariate COX analysis were included in the multivariate analysis.

All statistical tests were double-tailed tests, and the *p*<0.05 was considered to be statistically significant.

## 3 Result

### 3.1 Comparison of clinicopathological features between HCC Patients with normal and elevated serum CA19-9 levels after immunotherapy

**Figure 1** presents the flowchart of patient enrolment. According to the inclusion and exclusion criteria, we included 326 patients in this study. Of the 326 patients, after immunotherapy, 66 and 260 exhibited elevated and normal serum CA19-9 levels, respectively (hereafter referred to as the elevated and normal groups, respectively). **Table 1** summarizes the baseline characteristics of the two groups. All the listed variables were comparable between these groups (all *P* > 0.05).

**Table 1:** Comparison of clinicopathological factors between patients with CA19-9 (+) and CA19-9 (-).

Characteristics	CA19-9≥35U/mL,n=66	CA19-9<35U/mL,n=260	*P*-value
Age, years, means(SD)	57.2 (12.2)	54.3 (11.0)	0.063
Gender, n(%)			0.781
Female male	8 (12.1) 58 (87.9)	26 (10.0) 234 (90.0)	
MTD, cm, median(SD)	8.2 (52.5)	7.4 (4.7)	0.211
Tumor, n (%)			0.069
Solitary Multiple	12 (18.2) 54 (81.8)	79 (30.4) 181 (69.6)	
PVTT, n (%)	27 (40.9)	98 (37.7)	0.735
Hepatitis, n (%)			0.248
HBV HCV Other	57 (86.4) 0 (0) 9 (13.6)	231 (88.8) 6 (2.3) 23 (8.9)	
Liver Cirrhosis, n (%)	25 (37.9)	120 (46.2)	0.285
Extrahepatic metastasis,n (%)	23 (34.9)	58 (22.3)	0.052
ALB, g/L, median [IQR]	35.1 [32.3, 38.3]	36.2 [33.0, 39.0]	0.145
TBIL, umol/L, median [IQR]	21.5 [15.7, 29.7]	18.8 [13.7, 25.7]	0.103
PT, sec, median [IQR]	12.6[12.0-13.2]	12.7 (11.8-13.5)	0.774

CA19-9, carbohydrate antigen 19-9; SD, standard deviation; IQR, interquartile ranges; MTD, maximal tumor diameter; PVTT, portal vein tumor thrombus; HBV, hepatitis B virus; HCV, hepatitis C virus; ALB, serum albumin; TBIL, serum total bilirubin; PT, prothrombin time.

### 3.2 Comparison of prognosis between the normal and elevated CA19-9 groups

The median follow-up periods of the elevated and normal CA19-9 groups were 17.4 and 19.93 months, respectively. A total of 80 patients died during the follow-up. Kaplan–Meier analysis revealed that the median progression-free survival periods of the elevated and normal CA19-9 groups were 5.47 months (95% confidence interval [CI]: 3.96–6.98) and 13.23 months (95% CI: 11.30–15.16), respectively. The corresponding 1-year and 2-year PFS rates were 16.9% and 11.3% in the elevated CA19-9 group and 53.3% and 29.1% in the normal CA19-9 group (*P* < 0.001, **Figure 1B**). These results indicated that the elevated CA19-9 levels were positively correlated with shorter PFS in the patients with HCC receiving immunotherapy.

The median overall survival (OS) periods in the elevated and normal groups were 16.60 months [95% CI: 12.42–20.78] and 37.23 months [95% CI: 30.34–44.12], respectively. The corresponding 1-year and 2-year OS were 64.0% and 36.5% in the elevated group and 90.5% and 75.5% in the normal group, respectively (*P* < 0.001, **Figure 1C**). Survival analysis revealed that the elevated serum CA19-9 levels after immunotherapy were associated with poor survival.

### 3.3 Univariate and multivariate analyses

**Table 2** lists the predictors of PFS and OS identified through univariate and multivariate analyses. Univariate analysis revealed that CA19-9 levels (*P* < 0.001), extrahepatic metastasis (*P* = 0.001), and number of tumors (*P* = 0.020) were all significantly associated with PFS, whereas CA19-9 levels (*P* < 0.001), extrahepatic metastasis (*P* < 0.001), and PVTT (*P* = 0.049) were significantly related to OS. Multivariate analysis indicated that CA19-9 levels (HR: 2.68, 95% CI: 1.94–3.72, *P*< 0.001), extrahepatic metastasis (HR: 1.71, 95% CI: 1.26–2.32, *P* < 0.001), and number of tumors (HR: 0.69, 95% CI: 0.50–0.95, *P* = 0.022) were the significant prognostic factors for PFS. Furthermore, CA19-9 (HR: 3.31, 95% CI: 2.06–5.32, *P* < 0.001), HCC extrahepatic metastasis (HR: 2.64, 95%CI: 1.69–4.14, *P* < 0.001), and PVTT (HR: 1.67, 95% CI: 1.07–2.62, *P* = 0.024) were the significant prognostic factors for OS.

**Table 2:** Univariable and multivariable analysis of prognostic factors.

Variable	Progression-free Survival						Overall Survival					
	Univariable Analysis			Multivariable Analysis			Univariable Analysis			Multivariable Analysis		
	HR	95%CI	*P*-value	HR	95%CI	*P*-value	HR	95%CI	*P*-value	HR	95%CI	*P*-value
Age(≤60years)	1.06	0.78-1.44	0.690				0.85	0.53-1.35	0.492			
Gender(Male)	0.65	0.41-1.01	0.057				0.61	0.30-1.22	0.158			
MTD(≤5 cm)	0.93	0.67-1.28	0.637				0.84	0.50-1.42	0.496			
Tumor Number(Solitary)	0.69	0.50-0.94	**0.020**	0.69	0.50-0.95	**0.022**	0.70	0.43-1.15	0.163			
PVTT	1.14	0.86-1.52	0.367				1.56	1.00-2.42	**0.049**	1.67	1.07-2.62	**0.024**
Hepatitis (HBV/HCV/Other)	1.20	0.76-1.90	0.424				1.35	0.64-2.84	0.424			
Liver Cirrhosis	0.84	0.63-1.11	0.215				0.87	0.56-1.37	0.559			
Extrahepatic metastasis	1.67	1.23-2.25	**0.001**	1.71	1.26-2.32	**<0.001**	2.60	1.67-4.05	**<0.001**	2.64	1.69-4.14	**<0.001**
ALB(≤35g/L)	1.10	0.83-1.47	0.509				1.31	0.84-2.06	0.235			
TBIL(≤34 umol/L)	0.88	0.64-1.21	0.429				1.19	0.71-2.02	0.509			
CA19-9(≥35U/mL)	2.78	2.01-3.85	**<0.001**	2.68	1.94-3.72	**<0.001**	3.37	2.11-5.38	**<0.001**	3.31	2.06-5.32	**<0.001**

HR, hazard ratio; CI, confidence interval; MTD, maximal tumor diameter; PVTT, portal vein tumor thrombus; HBV, hepatitis B virus; HCV, hepatitis C virus; ALB, serum albumin; TBIL, serum total bilirubin; CA19-9, carbohydrate antigen 19-9. The bold text means P<0.05.

### 3.4 Relationship between *FUT3* expression and immune infiltration

**Figure 2:**
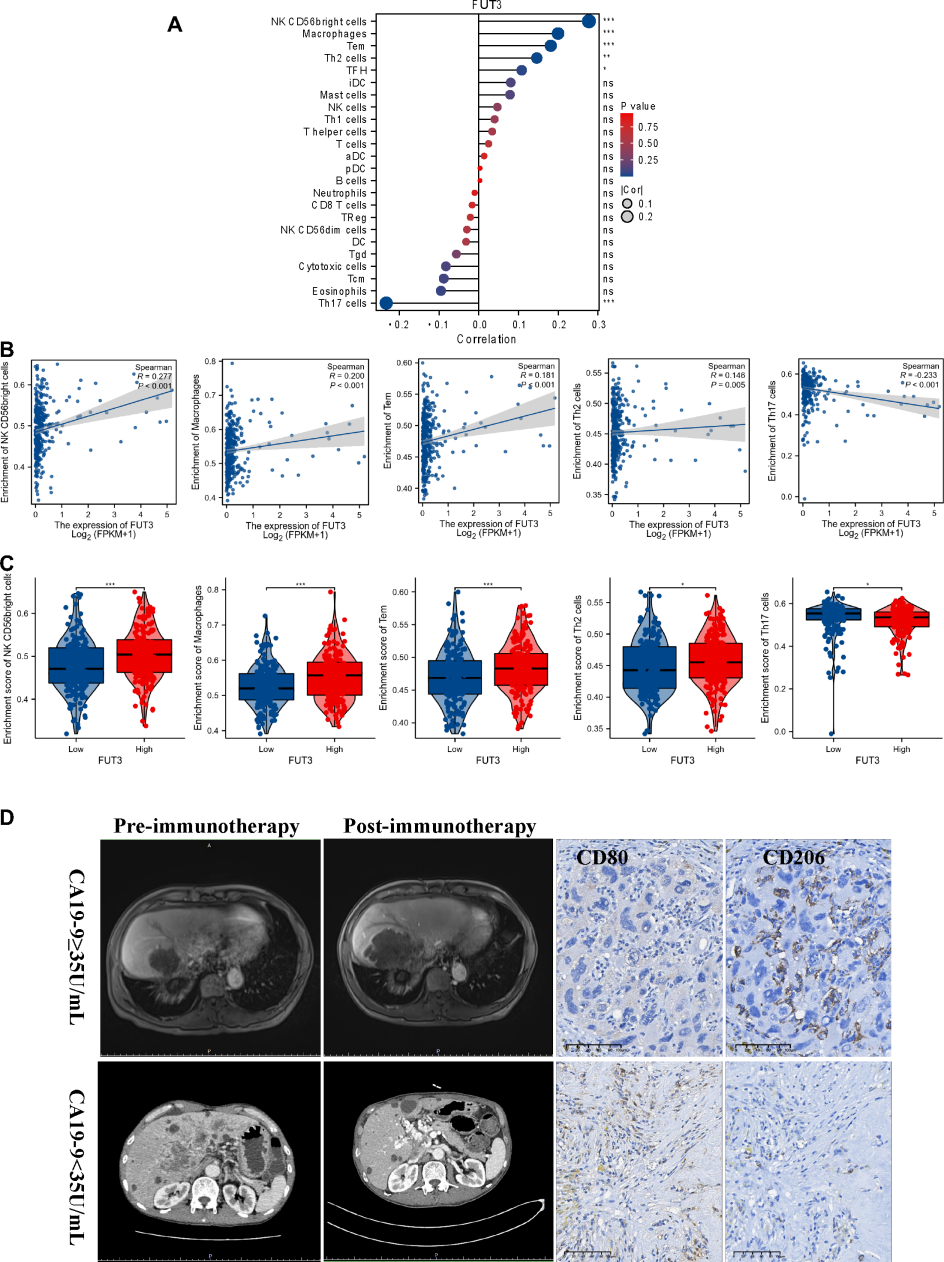
Results of correlation between *FUT3* expression and immune cell infiltration. **(A)** Gene Set Enrichment Analysis (GSVA) of the expression level between *FUT3* and the relative abundance of 24 types of immune cells. The bubble size represents Spearman’s rank correlation coefficient. **(B)** Correlation between the expression level *FUT3* and the relative enrichment scores of immune cells (including NK CD56 bright cells, macrophages, Tem, Th2 cells, and Th17 cells). **(C)** Spearman’s correlation analysis of the immune infiltration level of immune cells (including NK CD56 bright cells, macrophages, Tem, Th2 cells, and Th17 cells) in the high and low *FUT3* expression groups. **(D)** Representative MRI images of patients with HCC with or without elevated CA19-9 levels before/after immunotherapy (left panel). Representative immunohistochemistry images (CD80 and CD206) of patients with HCC with or without elevated CA19-9 levels after immunotherapy (right panel). CD80, a key marker for M1 macrophages; CD206, a key marker for M2 macrophages.

PD1/PD-L1 inhibitors enhance the immune response against cancer cells, and CA19-9 has been recognized as a potential prognostic factor in immunotherapy. We investigated the effect of CA19-9 on the tumor immune microenvironment. We determined the expression of *FUT3*, which is responsible for the synthesis of CA19-9 (22), and evaluated the infiltration of various immune cells in HCC tissues through the single-sample gene set enrichment analysis (**Figure 2A**). We assessed the infiltration of 24 types of immune cells in HCC tissues and observed correlations between the expression levels of *FUT3* and the infiltration of specific immune cell subtypes (**Figure 2A**). Spearman correlation analysis was performed to investigate the correlation between the expression level of *FUT3*, measured in transcripts per million format, and the infiltration level of immune cells. The results demonstrated that the infiltration of NK CD56 bright cells (Spearman r = 0.277, *p* < 0.001), macrophages (Spearman r = 0.200, *p* < 0.001), Tem (Spearman r = 0.181, *p* 0.001), and Th2 cells (Spearman r = 0.146, *p* = 0.005) was positively correlated with *FUT3* expression (**Figure 2B**), with markedly higher infiltration observed in the group exhibiting high *FUT3* expression (**Figure 2C**). By contrast, we noted a negative correlation between *FUT3* expression and Th17 cell infiltration (Spearman r = −0.233, *p* < 0.001). Macrophage infiltration plays a vital role in HCC (23–25). **Figure 2D** presents the typical computed tomography (CT) and magnetic resonance imaging (MRI) images of patients with HCC with normal or elevated serum CA19-9 levels before and after immunotherapy. Given the crucial role of macrophages in HCC, we investigated how CA19-9 levels and immunotherapy affect macrophage infiltration and polarization. We observed a substantial increase in the density of CD206+ tumor-associated macrophages (TAMs, indicative of M2 macrophages) in HCC samples with elevated CA19-9 levels compared with those without elevated CA19-9 levels post immunotherapy. However, the level of CD80+ TAMs (M1 macrophages) was lower in HCC samples with elevated CA19-9 levels than in those without elevated CA19-9 levels (**Figure 2D**). Altogether, the increased prevalence of M2 macrophages in HCC samples with elevated CA19-9 levels sheds light on potential implications for the prognosis of patients with HCC receiving immunotherapy.

### 3.5 CA19-9 blocker improves anti-PD1 efficacy by inducing M1-like polarization of macrophages

**Figure 3:**
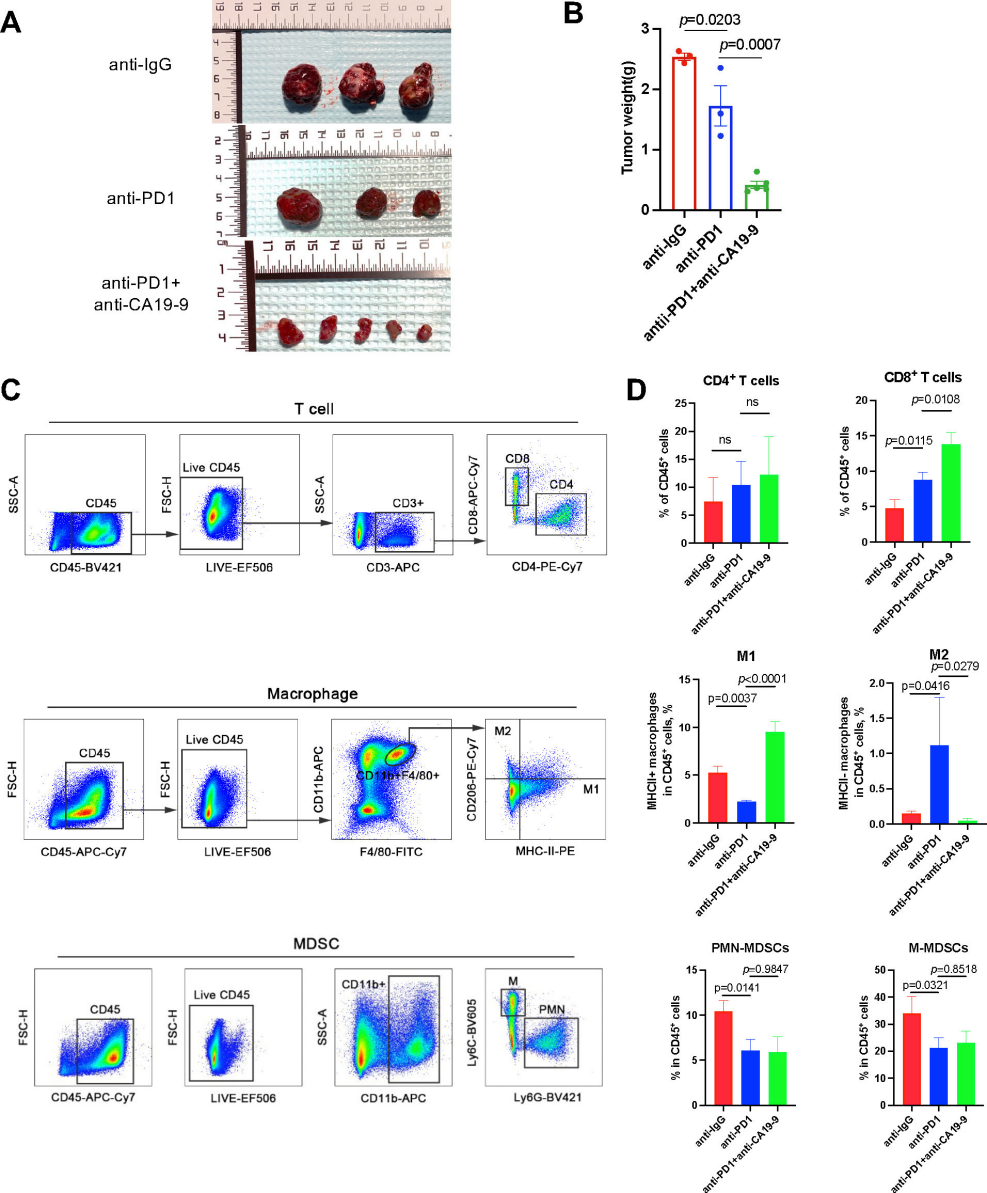
Blocking CA19-9 improves anti-PD1 efficacy by inducing M1-like polarization of macrophages. Wild-type C57BL/6 mice inoculated with subcutaneous Hepa1-6 cells were administered 200 μg of the anti-PD1 antibody (intraperitoneally every three days) with or without 200 μg of anti-CA19-9 (intraperitoneally daily) after they developed palpable tumors (after 2 weeks) prior to tumor evaluation. **(A)** Representative macroscopic appearance of tumors at the experimental endpoint. **(B)** Tumor weight. **(C)** Gating strategy for each cell type of T cells, macrophages, and MDSCs from single-cell suspensions derived from Hepa1-6 tumors in C57BL/6 mice. **(D)** Frequency of each cell type of T cells, macrophages, and MDSCs in total CD45+ cells in the anti-IgG, anti-PD1, and anti-PD1 + anti-CA19-9 groups. *p < 0.05 versus the cohort of control mice, one-way analysis of variance with post Tukey’s comparison test for **(B, D)**.

To evaluate the effect of a CA19-9 inhibitor on HCC and the tumor immune microenvironment, we used a subcutaneous xenograft tumor model with Hepa1-6 cells. Our findings revealed that the treatment with an anti-PD1 antibody significantly reduced tumor growth compared with treatment with an anti-IgG antibody. The addition of an anti-CA19-9 antibody further suppressed tumor proliferation (**Figures 3A, B**). To understand how CA19-9 inhibition affects HCC, we analyzed the tumor immune microenvironment through flow cytometry. Macrophages identified as CD11b+F4/80+ within the CD45+ cell population were categorized into two groups based on MHCII expression levels, similar to observations in a colon adenoma model (26). Our results demonstrated an increase in M2-polarized macrophages and a decrease in M1-polarized macrophages in tumors treated with anti-PD1 compared with those treated with IgG. This effect was reversed following the administration of an anti-CA19-9 antibody (**Figures 3C, D**). In addition, the combination of anti-CA19-9 therapy with or without anti-PD-1 significantly enhanced the tumor infiltration of CD8+ T cells and reduced the presence of myeloid-derived suppressor cells (MDSCs), including both polymorphonuclear (PMN)-MDSCs and mononuclear (M)-MDSCs (**Figures 3C, D**).

## 4 Discussion

The approval of anti-PD/PD-L1 therapy by the US Food and Drug Administration has significantly advanced HCC treatment, establishing a new clinical standard and potential foundation for immune combination therapies. However, not all patients with HCC benefit from immunotherapy, highlighting the need for the identification of biomarkers to select suitable candidates. CA19-9, a serum marker often synthesized by the bile duct epithelium in the hepatobiliary system, is easily accessible in clinical settings. This marker has been associated with several cancers, including pancreatic, bile duct, colorectal, and gastrointestinal tumors (27–29). In this study, we explored the role of CA19-9 as a prognostic factor for patients with HCC undergoing immunotherapy. We observed that elevated serum CA19-9 levels after immunotherapy were associated with a poor prognosis. Furthermore, the expression levels of macrophages were found to be positively correlated to those of *FUT3*, a key gene involved in CA19-9 synthesis, indicating a significant correlation with the tumor microenvironment. In addition, our findings demonstrated that the inhibition of CA19-9 improved anti-PD1 efficacy *in vivo* by inducing M1-like polarization of the macrophages.

Macrophage polarization, particularly the balance between M1 and M2 phenotypes, plays a critical role in shaping the tumor microenvironment (TME) and influencing therapeutic outcomes in HCC. Based on our findings and existing literature, we propose the following potential mechanisms underlying the differential macrophage polarization observed: Firstly, elevated CA19-9 levels may promote the secretion of cytokines and chemokines (e.g., CCL2, CXCL8), which attract monocytes to the TME (30–32). These recruited monocytes are subsequently exposed to the TME’s immunosuppressive signals, favoring M2-like differentiation. Secondly, CA19-9 may enhance the interaction between sialylated glycans and inhibitory receptors such as Siglec-5 and Siglec-10 on macrophages (14). This interaction suppresses pro-inflammatory responses and skews macrophages towards the M2 phenotype. CA19-9 may also upregulate TGF-β and IL-10 expression in the TME, further driving M2 polarization (33). Thirdly, M2 macrophages in HCC secrete factors like VEGF and IL-10, which inhibit effector T-cell infiltration and function, creating an immunosuppressive TME (34, 35). And these macrophages also express high levels of PD-L1, directly dampening T-cell activation and further reducing the efficacy of ICIs. Last but at least, our study demonstrated that blocking CA19-9 reduces inhibitory signaling in macrophages, leading to a shift towards M1-like polarization. And M1 macrophages are associated with enhanced antigen presentation, increased secretion of pro-inflammatory cytokines (e.g., TNF-α, IL-12), and improved recruitment and activation of CD8+ T cells, collectively promoting anti-tumor immunity. These potential mechanisms underscore the pivotal role of CA19-9 in modulating macrophage behavior and the broader immune landscape in HCC.

Previous studies have reported that serum CA19-9 levels were associated with a poor prognosis of various tumors, including pancreatic cancer (27), bile duct cancer (29), colorectal cancer (28), and other gastrointestinal tumors. For liver cancer, an increase in serum CA19-9 levels before surgery (surgical resection or liver transplantation) and before local treatments (e.g., radiofrequency ablation) is associated with poor survival outcomes for patients with HCC (9, 10, 36, 37). Despite the observed increase in serum CA19-9 levels in patients with HCC, its predictive value and role, especially in the context of immunotherapy, remain unclear. In this study, we analyzed the CA19-9 level in patients with HCC after immunotherapy and found that CA19-9 can serve as a prognostic factor for patients with HCC receiving immunotherapy. An increase in CA19-9 levels indicated poorer survival outcomes for patients with HCC treated with ICIs, affecting both PFS and OS (**Figures 1B, C**). Furthermore, CA19-9 has been shown to facilitate tumor progression by acting as a selection ligand that promotes tumor adhesion and mediates blood-borne metastasis (38, 39) as well as leads to tumor angiogenesis (40). In the present study, we determined that blocking CA19-9 improves the efficacy of anti-PD1 therapy in a mouse model of HCC, indicating that targeting CA19-9 might enhance the efficacy of immunotherapy in HCC (**Figures 3A, B**).

Therapeutic antibodies targeting CA19-9 were reported to successfully induce antitumor immune responses (41), and CA19-9 monoclonal antibodies demonstrated antibody-dependent cell-mediated cytotoxicity in mouse experiments (42). Our findings are consistent with those of previous studies that have highlighted the potential of therapeutic antibodies aimed at CA19-9 to elicit antitumor immune responses. CA19-9 may possess the ability to reshape the tumor microenvironment, leading to varied responses to ICIs in HCC. Bioinformatic analysis using TCGA database revealed that the expression of *FUT3* (a key gene involved in the synthesis of CA19-9) is positively correlated with the infiltration of NK CD56 bright cells, macrophages, Tem, and Th2 cells and negatively correlated with Th17 cell infiltration (**Figure 2**). Immunohistochemistry analysis of human HCC samples with normal or elevated serum CA19-9 levels indicated an increase in the level of M2 macrophages in HCC samples with elevated CA19-9 levels. Moreover, anti-PD1-treated tumors exhibited a higher number of M2-polarized macrophages and a lower number of M1-polarized macrophages than IgG-treated tumors (**Figures 3C, D**). This phenomenon was reversed following the administration of anti-CA19-9. Altogether, these findings indicate that CA19-9 might affect the efficacy of immunotherapy by influencing the polarization of macrophages infiltrated in the tumor microenvironment.

This study has several limitations that warrant acknowledgment. First, the non-prospective, non-randomized design has inherent limitations, including potential selection bias that cannot be fully avoided. Second, limited baseline data made it difficult to completely eliminate sample heterogeneity, potentially affecting the generalizability of the findings. Last, CA19-9 is expressed in various tumor types, and prior studies have demonstrated its association with poor prognoses in multiple cancers. However, whether the findings of this study can be generalized to other malignancies remains uncertain and warrants further investigation. These limitations underscore the need for future prospective, randomized studies with more comprehensive data collection and analysis to validate and extend these findings in HCC and other malignancies.

In conclusion, our study suggests that CA19-9 serves as a promising prognostic marker for patients with HCC receiving immunotherapy. Elevated CA19-9 levels following immune checkpoint therapy were associated with poor survival outcomes in HCC, and anti-CA19-9 therapy may enhance the efficacy of immunotherapy. Therefore, we conclude that inhibiting CA19-9 may benefit HCC patients with elevated serum CA19-9 levels and poor responses to immune checkpoint therapy. The clinical efficacy of combining CA19-9 inhibition with ICIs requires further research to be confirmed.

## Data Availability

The raw data supporting the conclusions of this article will be made available by the authors, without undue reservation.
